# Motivational Dynamics in a Multilingual Context: University Students’ Perspectives on LOTE Learning

**DOI:** 10.3390/bs15070931

**Published:** 2025-07-10

**Authors:** Ali Göksu, Vincent Louis

**Affiliations:** 1 Department of Educational Development and Research, Faculty of Health, Medicine, and Life Sciences, School of Health Professions Education (SHE), Maastricht University, 6200 MD Maastricht, The Netherlands; 2 Centre de Recherche Tradital, École de Traduction et Interprétation (ISTI-Cooremans), Université Libre de Bruxelles, 1050 Bruxelles, Belgium; vincent.louis@ulb.be

**Keywords:** motivation, LOTE, additional language, multilingual learners

## Abstract

Interest in language-learning motivation has been growing recently, particularly in multilingual contexts where individuals acquire additional languages beyond English. Despite increasing the focus on multilingualism within second-language acquisition (SLA) research, less research focuses on the motivational dynamics of multilingual learners in learning languages other than English (LOTE). Addressing this gap, the present study investigates the complex motivational factors influencing multilingual university students in learning French as an additional language and LOTE within the Belgian context. The participants consisted of 121 multilingual university students who were learning French as an additional language and LOTE. Data were collected through questionnaire and semi-structured interviews, and analyzed using a combination of quantitative and qualitative methods to provide a comprehensive understanding of learners’ motivational profile. Findings revealed that multilingual learners’ motivation is multifaceted and dynamic, shaped by a combination of intrinsic interests (e.g., cultural appreciation and personal growth), extrinsic goals (e.g., academic and career aspirations), integrative motives, and prior language-learning experiences. The study also sheds light on the overlapping and evolving nature of motivational patterns and provides nuanced insights into LOTE learning motivation within multilingual settings.

## 1. Introduction

In recent years, language-learning motivation has drawn many researchers’ attention with its complex and dynamic aspects in the field of second-language acquisition (SLA) (Dörnyei, 2009; Gardner, 2010; Henry, 2011; Lai, 2023; Thompson & Lee, 2018). In addition, a growing number of studies have recently investigated new ideas and concepts emerging around the phenomenon of multilingualism (Cohen & Kassis-Henderson, 2017; Cenoz, 2013; Jessner, 2008) to take the “multilingual turn” (May, 2014; Ortega, 2014), providing broad perspectives on research in second-language (L2) learning (Ushioda, 2021). While research on language-learning motivation is popular in the domain of SLA studies, a limited number of studies focus on the relationship between motivation and multilingualism (Csizér & Lukács, 2010; Henry & Thorsen, 2018; Thompson & Lee, 2018; Thompson & Liu, 2021). Moreover, although the majority of empirical research has focused exclusively on motivation to learn English (Boo et al., 2015), less studies have explored the individual’s motivation to learn languages other than English (LOTEs), or additional language in multilingual environments (D’Orazzi & Hajek, 2022; Lai, 2023; Siridetkoon & Dewaele, 2018; Ushioda & Dörnyei, 2017). Furthermore, the motivations of individuals—particularly multilingual learners—are shaped by a complex interplay of factors, including intrinsic interest, the pursuit of external rewards (Self-Determination Theory) (Deci & Ryan, 1985), or prior language-learning experiences (L2 Motivational Self System) (Dörnyei, 2005). While these offer valuable insights, it is still unclear, from a broader perspective, what motivations drive multilingual university students to learn an additional language or LOTE. Addressing these gaps, the present study examines various motivational constructs of multilingual university students in learning French as an additional language and LOTE within the multilingual context of Belgium. This research further provides a comprehensive perspective on multilingual learners’ motivation in learning LOTE, with a particular focus on university students from predominantly European backgrounds.

## 2. Literature Review

### 2.1. Language-Learning Motivation and Multilingualism

Motivation, as a driving force, key component and an important phenomenon for successful foreign/second-language (FL/L2) learning, has attracted the attention of students, teachers, and researchers in recent decades (Dörnyei, 2009; Lamb et al., 2019; Thompson & Erdil-Moody, 2016). While the construct of motivation in the context of language learning has been investigated through the lens of various approaches and models, “no single account or approach can ever hope to suffice” because of its multifaceted nature (Ryan, 2019, p. 178). The most common motivation models used in FL/L2 learning are the socio-educational model (Gardner, 1985, 2010), self-determination theory (SDT) (Deci & Ryan, 1985; Ryan & Deci, 2017), and L2 Motivational Self-System (L2MSS) (Dörnyei, 2005, 2009). Gardner’s (1985, 2010) socio-educational model entails integrative versus instrumental motivation for L2 learning, whereas SDT (Ryan & Deci, 2002) focuses mainly on language learners’ intrinsic and extrinsic motivation in L2 learning. Dörnyei’s L2MSS (Dörnyei, 2005, 2009) is a theoretical model which describes motivation and consists of three main components: the ideal L2 self (the learner’s internal desire to become an effective L2 user), the ought-to L2 self (social pressures and external influences coming from the learner’s environment to master the L2), and the L2 learning experience (the actual experience of being engaged in the L2 learning process) (Dörnyei & Al-Hoorie, 2017). Taken together, in addition to similar perspectives, each model has distinct characteristics by taking into account language learners’ motives arising from within individuals or being influenced from outside in describing language motivation.

On the other hand, due to the increase in immigration and globalization across the world, the “multilingual turn” (Ortega, 2014, p. 33) has triggered a shift in L2 research exploring novel ideas and concepts relating to multilingualism (Jessner, 1999; Cenoz, 2013; May, 2014). Multilingual people have more advantages over monolinguals in cognitive development, metalinguistic awareness, divergent thinking, imagination, grammatical awareness, perceptual organization, reading achievement, and heightened sensitivity in communicative skills (Cenoz, 2003, pp. 73–74). Research suggests that having a multilingual background is beneficial for language learners, as they are able to deploy more effective language-learning strategies (Cenoz, 2013; Cummins, 2007), score higher on language aptitude tests (Thompson, 2013), be more tolerant (Dewaele & Wei, 2013; Thompson & Erdil-Moody, 2016; Thompson & Lee, 2018), and experience less anxiety (Dewaele et al., 2008).

Being multilingual is itself a source of motivation to learn L2 or additional languages (Busse, 2017; Costache et al., 2022; Dörnyei & Al-Hoorie, 2017; Lasagabaster, 2017). Although many studies investigated the relationship between the motivation and a single second or foreign language, there has been relatively less research on motivation and multilingualism (Henry, 2011, 2017; Lasagabaster, 2017; Thompson & Erdil-Moody, 2016; Thompson & Liu, 2021). For instance, Henry (2011) investigated how L2 and L3 (third-language) motivational constructs relate to one another and found that L2 motivation can indeed impact L3 motivation. In another research paper, Thompson and Erdil-Moody (2016) examined differences between bilinguals and multilinguals with regard to their ideal and ought-to L2 selves on motivation. The findings showed that the ideal L2 selves of multilinguals are significantly different from those of other groups of learners, when using both a more traditional definition of multilingualism (i.e., experience with multiple languages), as well as an innovative way of operationalizing multilingualism.

### 2.2. Motivation Beyond L2 Learning

Research on motivation in L2 literature has extensively focused on motivation pertaining to one specific second or foreign language (Boo et al., 2015; Dörnyei & Al-Hoorie, 2017; Ren & Wang, 2025; Thompson & Lee, 2018; Ushioda & Dörnyei, 2017). However, motivation in third- or additional-language learning is assumed to be even more changeable, as it is more complex than L2 learning, especially when learners consider the additional language as interesting and useful but do not believe that learning it is absolutely necessary (Włosowicz, 2013). According to Marten and Mostert (2012), additional language acquisition is a particularly relevant concept for the teaching and learning of languages of the wider world or less widely taught languages since, at least in the European context, these are often learned by learners who already have experience of learning more widely taught languages, such as French, German, Spanish, or English (p. 101). In addition, learners who have acquired two or more additional languages could develop an ideal multilingual self—a motivational construct that can positively influence their drive to learn further languages (Henry, 2017). This concept aligns with the view that prior language-learning experiences play a facilitative role in the acquisition of additional languages (Dörnyei, 2005, 2009). Research further indicates that previously acquired languages could positively influence the learning of additional languages and enhance multilingual individuals’ capacity to acquire additional languages (Thompson & Erdil-Moody, 2016; Thompson & Lee, 2018).

Numerous studies have reported that a multilingual background may enhance learners’ motivation to acquire additional languages (Cenoz, 2003; Costache et al., 2022; Dewaele, 2010). However, there has been less research on additional language or L3 motivation in multilingual settings (Bui et al., 2018; Costache et al., 2022; Marten & Mostert, 2012; Thompson & Lee, 2018). For example, Marten and Mostert (2012) investigated university students’ linguistic background, their motivation and reasons, and their self-assessed progress for studying Zulu as an additional or L3 language in higher education in the UK. The results indicated that participants had mostly integrative and instrumental (extrinsic) motivation, such as personal, academic, and professional reasons for studying Zulu in South Africa. In another study examining motivation from both an L2 (English) and L3 (Japanese) perspective, with a focus on motivational similarities and differences, Bui et al. (2018) found that students developed distinct motivational profiles. Their L2 (English) learning was primarily driven by extrinsic factors, whereas their L3 (Japanese) learning was characterized by stronger cultural interest, positive learning experiences, greater self-confidence, and more favorable attitudes.

Recent research has also explored how diverse language-learning experiences—shaped by regional and sociolinguistic contexts— influence the development of language-learning motivation, particularly in multilingual environments where learners navigate multiple languages with varying status and personal relevance (Bui et al., 2018; Costache et al., 2022; Wu & Liu, 2023). For instance, Costache et al. (2022) focused on the longitudinal relations between Swiss German students’ value beliefs in English, French, and German, as well as the differences in motivational development between multilingual and monolingual students in Switzerland, a historically multilingual country. The findings revealed that students who reported higher value beliefs in English showed a steep decrease in their value beliefs for French and German. In addition, multilingual students reported higher initial value beliefs in French and English, and also showed steeper decreases in French and English value beliefs over time compared to their monolingual peers. These findings underscore the complex and dynamic nature of multilingual learners’ motivation, highlighting the influence of both individual language experiences and broader sociolinguistic contexts in shaping motivational trajectories.

In addition to these influences, individual learner differences such as gender and language proficiency have also been identified as important factors shaping learners’ motivation and attitudes toward the target (additional) language. Some research has found that females have been more motivated than males in L2 learning (Dörnyei & Csizér, 2002; Henry, 2009), whereas others indicated that there were no significant differences for gender (Henry & Cliffordson, 2013; Sylvén & Thompson, 2015; Thompson & Erdil-Moody, 2016). With regard to the relationship of motivation to proficiency, research shows that there has generally been a significant difference between the lower- and advanced-level groups (Kim, 2012; Thompson & Erdil-Moody, 2016).

### 2.3. Motivation for Learning LOTEs

The majority of research on motivation in L2 literature has focused on the learning of English because of its dominant status as a global language across the world (Boo et al., 2015; Busse, 2017; Huang et al., 2021; Ushioda & Dörnyei, 2017). The motivation for learning languages other than English (LOTEs) is also salient for learners who are concerned with promoting, supporting, and enhancing language learning beyond global English (Ushioda & Dörnyei, 2017). LOTEs are predominantly learnt as an L3 or additional language following on from English as the first language learnt as an L2, while LOTEs are sometimes learnt as L2 by the English native speakers (Howard & Oakes, 2021, p. 2). Although English has received extensive attention in language learning-motivation research due to its international status across the world, limited research has explored learners’ motivation to learn other languages classified as a LOTE (Göksu & Louis, 2024; Huang et al., 2021; Lai, 2023; Ushioda & Dörnyei, 2017; Wu & Liu, 2023). For instance, Boo et al. (2015) reported in their study reviewing a large body of research published between 2005 and 2014 on L2 motivation that English as a target language was very dominant in the studies during this period, and 72.6% of empirical research was conducted to explore the motivation in learning English as L2. Huang et al. (2021) also investigated the potential emergence of a multilingual motivational system in the E-LOTE learners and compared Chinese English+LOTE (E-LOTE) learners to English-only learners. Their study revealed that E-LOTE learners had a higher motivation to learn English at the beginning and during their development. Moreover, the E-LOTE learners’ motivation to learn the two languages interacted with each other over time. In another research, Wu and Liu (2023) explored the L3 motivational dynamics of four Japanese-major university students as LOTE speakers in China through in-depth narrative interviews. They found that learners’ meaning-making of experience provided the foundation for their self-guide construction, which led to the emergence of motivation. It is also worth noting that some people prefer learning LOTE because they have insufficient competence in L2 English, whereas others may consciously choose a LOTE as an additional language since it provides them a competitive benefit in the job market (Siridetkoon & Dewaele, 2018). Furthermore, researchers underline that there is still a gap between the motivation and LOTE as additional language in the literature (Boo et al., 2015; Dörnyei & Al-Hoorie, 2017; Dörnyei & Ushioda, 2021; Ushioda, 2017; Ushioda & Dörnyei, 2017).

### 2.4. Context

The Council of Europe (2008, p. 566) suggests that each citizen in Europe should be proficient in three European languages, known as the “1 plus 2” model, which allows citizens to communicate in two additional languages plus their mother tongue. In addition to being a multilingual speaker as an essential characteristic feature of European identity, speaking fluently in more than one foreign language has currently become a major and current educational goal in Europe (Council of Europe, 2019). Belgium is also situated within a multilingual context in Europe with its three official languages (French, Dutch, and German) and an important context with its linguistic communities for investigations into the effects of the social context (Dewaele, 2005). As a capital city of Belgium and the European Union, Brussels is the hub of a very diverse multilingual and multicultural community across the world. Today, beyond the FL/L2 learning, globalization, immigration, and professional mobility continue to add to the linguistic and cultural diversity of Brussels. In Brussels, where a wide range of world languages are spoken fluently as additional languages, French currently holds a dominant position as a first language (Ceuleers, 2008). The present study was conducted at the Université libre de Bruxelles (ULB), located in Brussels, where French is the primary language of instruction for the majority of study programs. All participants in this study were actively engaged in degree programs delivered in French as the medium of instruction.

## 3. The Study

This research addresses several gaps in the current literature on L2 learning, multilingualism, and motivation. This study focuses on examining the various motivational constructs of multilingual university students learning French as LOTE and additional language. Previous studies on language-learning motivation have primarily focused on FL/L2 (English) motivation, with specific motivational constructs such as anxiety, intrinsic and extrinsic motivation, and interest across diverse contexts; however, limited research focuses comprehensively on motivation for learning additional language(s) beyond FL/L2 (Costache et al., 2022; Henry, 2011; Thompson & Lee, 2018). Additionally, it is not yet clear what motivations specifically drive multilingual students to learn French as LOTE (Boo et al., 2015; Dörnyei & Al-Hoorie, 2017; Ushioda & Dörnyei, 2017). Furthermore, although recent research on L2 motivation and multilingualism investigates the similarities and differences between L2 and L3 learning (Bui et al., 2018; Lai, 2023), there is still less research examining the motivation for an additional language or LOTE in multilingual environments. Addressing these gaps, this research explored various motivational constructs influencing multilingual university students learning French as additional language and LOTE in the context of Belgium. In this study, French is considered a LOTE and learned as an additional language (L3 or L4) by multilingual university students.

### Research Questions

(1) Are there significant differences among various motivational constructs (e.g., intrinsic and extrinsic motivation, interest in languages and culture, expectancy, language aptitude, anxiety, etc.) influencing multilingual university students when learning French as an additional language and LOTE?

(2) Do motivational constructs for learning French among multilingual university students differ based on language-proficiency levels and gender?

(3) What are the primary motivational factors driving multilingual university students to learn French as an additional language and LOTE?

## 4. Methods

### 4.1. Participants

This study employed snowball sampling (Kirchherr & Charles, 2018) to recruit multilingual university students who speak French as an additional language and LOTE. While this approach was effective in reaching a specific target population, it may have contributed to an unbalanced sample in terms of gender and overall distribution. A total of 144 multilingual university students initially participated in the study. However, native or bilingual French speakers (n = 23) were excluded from the dataset to focus on learners of French as an additional language (L3, L4). The final sample consisted of 121 international university students (35 male, 86 female), aged 17 to 35 years (M = 23.41, SD = 3.96). Participants were enrolled in undergraduate (bachelor’s degree, n = 61) and postgraduate (master’s and PhD, n = 60) programs across various departments—including languages and letters, economics, engineering, and psychology—at the Université libre de Bruxelles (ULB) in Belgium. French was the medium of instruction for all participants, who were pursuing university study programs. Additionally, all participants were multilingual students who could speak three or more languages, and French was an additional language (L3 or L4) and LOTE for all participants. They all self-reported the additional languages they could speak and their proficiency levels. The majority of participants (n = 67) reported proficiency in two additional languages, including French, excluding their mother tongues. Other participants also indicated proficiency in three (n = 36), four (n = 16), five (n = 1), or even six (n = 1) additional languages—also excluding their mother tongues—with French included among them. Participants also self-rated their French proficiency using the Common European Framework of Reference for Languages (CEFR) levels as follows: A2 (Elementary; n = 17), B1 (Intermediate; n = 46), B2 (Upper-Intermediate; n = 45), and C1 (Advanced; n = 13). Some participants also provided their DELF or DALF test scores for French. Table 1 presents the participants’ mother tongues/first language (L1), highlighting the linguistic diversity of the sample, which included students from 23 different countries. Most participants reported a European language as their first language (L1), with the most represented being Italian (n = 27), Turkish (n = 16), Spanish (n = 15), and German (n = 13). All participants provided informed consent prior to data collection. Participation was both voluntary and anonymous.

### 4.2. Measurements

Data were collected through a questionnaire and semi-structured interview in the spring term of 2022–2023. The current study adopted a questionnaire designed for French to identify the different motivational factors in learning French as a LOTE. The questionnaire was originally developed by Schmidt and his colleagues (Schmidt et al., 1996) with a broader scope and different aspects of language-learning motivation, and it was later adapted and used by Hatcher (2000) and Balaman-Uçar (2009) in different contexts. The questionnaire contained 62 items pertaining to intrinsic motivation (6 items), extrinsic motivation (9), interest in languages and culture (5), integrative motivation (4), competitiveness (3), cooperativeness (4), the value of the language course (3), the belief in doing well or getting high grades (6), language aptitude (4), attitudes toward the target language (5), language anxiety (6), and items designed to gauge the learner’s intention to put their best effort into learning the language (7). The items of the questionnaire were originally drafted in English. All items were then translated into French and adapted to French. To assess reliability, Cronbach’s alpha was calculated for the questionnaire and yielded a coefficient of 0.86. All dimensions of the questionnaire demonstrated acceptable reliability (α ≥ 0.70) and internal homogeneity (*p* > 0.05). The questionnaire also included demographic and educational information in the form of 5-point Likert scales, from 1 (strongly agree) to 5 (strongly disagree).

The other instrument was a semi-structured interview, including a predetermined set of questions prepared by the researchers based on motivational dimensions in the questionnaire (e.g., “Why do you learn French? please give us some reasons?” and “Are you interested in learning other languages and cultures? Why or why not?”). This semi-structured interview was conducted to gain in-depth understanding of the multilingual university students’ motivations toward learning French as additional language and LOTE. All participants answered the interview questions in writing since they felt free to express their thoughts.

### 4.3. Data Analysis

Regarding the analyses, after descriptively examining the demographic details of multilingual university students, the data were quantitatively analyzed using SPSS 28, employing t-tests and one-way ANOVA. Additionally, comparisons were made to assess significant differences in gender, proficiency levels, and motivational constructs influencing participants’ motivation to learn French. Post hoc tests were conducted to identify specific differences between proficiency levels among French learners. Then, the responses given to the semi-structured interviews were qualitatively analyzed using Atlas.ti 25, providing thematic analysis through the coding of responses to identify and examine themes in the data (Braun & Clarke, 2006). Participants’ responses were examined individually to identify both overarching and condition-specific themes through a systematic multi-step coding procedure conducted independently by the researchers. The analysis process involved dividing the dataset into three portions (25%, 25%, and 50%, respectively). The first author coded the first dataset and refined the coding framework, while the second author followed the same procedure for the second dataset. Both authors collaboratively reviewed the codes from the first and second datasets to ensure consistency, resolving minor discrepancies through discussion and comparison of their coding. Reliability was reinforced at each stage of the analysis. The finalized coding framework was then applied by the first author to the remaining dataset. Finally, the first and second authors jointly reviewed all coded data to ensure overall consistency and accuracy. This thematic analysis provided deeper insights into the participants’ experiences of learning French and illuminated the motivational factors driving these multilingual French learners. Common themes and notable remarks were reported, with participant citations anonymized using numerical identifiers (e.g., P1, P2, and P3) to preserve confidentiality.

## 5. Results

### 5.1. Quantitative Findings: Variations in Various Motivational Constructs

Table 2 reveals the mean scores for each motivational construct differed significantly from each other. The findings also indicated that all motivational constructs were highly significant in driving motivation for learning French (*p* < 0.001), with effect sizes ranging from moderate to large (from d = 0.47 to d = 1.12) (Table 2). Interest in foreign language and culture (t(120) = 87.99, *p* < 0.001, d = 0.52) and cooperativeness (t(120) = 73.66, *p* < 0.001, d = 0.59) were among the highest-rated motivational constructs, highlighting participants’ strong motivation to engage with other languages and collaborate with others. Conversely, although competitiveness (t(120) = 27.14, *p* < 0.001, d = 1.12) and anxiety (t(120) = 37.04, *p* < 0.001, d = 0.81) had lower mean scores, both demonstrated strong effect sizes, suggesting that they remain meaningful motivational factors for learners.

For gender differences, independent sample t-tests’ results indicated no statistically significant differences between male and female participants across all measured motivational constructs (*p* > 0.05). With regard to the differences in motivational constructs on language proficiency levels, Table 3 indicates no statistically significant differences (*p* > 0.05) across most motivational constructs, including intrinsic motivation, extrinsic motivation, integrative motivation, interest in foreign languages and cultures, competitiveness, cooperativeness, task value, expectancy, attitude, and motivational strengths. However, we found significant differences in language aptitude (*p* = 0.013) and anxiety (*p* = 0.005). Furthermore, as shown in Table 3, the post hoc test (Bonferroni), which provided multiple comparisons between proficiency levels, revealed that the statistical differences were generally between lower (A2-Elementary) and higher proficiency (B2-Upper-Intermediate and C1-advanced) levels (*p* < 0.05). Learners’ perceptions of language aptitude also differed significantly (F(3, 117) = 3.716, *p* = 0.013), with A2-Elementary learners (M = 2.82, SD = 0.51) rating their aptitude significantly lower than B2-Upper-Intermediate (M = 3.40, SD = 0.68) and C1-Advanced learners (M = 3.51, SD = 0.68). Similarly, anxiety levels varied significantly across proficiency levels (F(3, 117) = 4.453, *p* = 0.005), with C1-Advanced learners (M = 2.30, SD = 0.59) reporting lower anxiety compared to A2-Elementary (M = 3.00, SD = 0.79) and B1-Intermediate learners (M = 2.93, SD = 0.84).

### 5.2. Quantitative Insights: Multilingual University Students’ Motives for Learning French

In regard to participants’ specific motivational factors for learning French, the qualitative analysis identified several significant themes and motivations, each playing a distinct role in learning French as an additional language and LOTE. Regarding the question about why they learn French, among extrinsic motivations, social integration emerged as a major theme, with many participants emphasizing the importance of learning French to connect with local communities, build relationships, and feel part of their environment. One participant remarked, “Because it’s important for me to communicate when I’m with French-speaking people” (P09), while another shared, “[French helps me] to be able to express myself in a French-speaking environment” (P53). Similarly, employability and career advancement were frequently mentioned, with learners viewing French as a valuable skill to access better job opportunities and excel professionally. A participant highlighted, “I’m doing the master’s in European studies because French is important for my future” (P76), while another noted, “The more languages you know the better possibilities you have in your job career” (P88). When viewed from the intrinsic motivation perspective, many participants expressed a deep appreciation for the beauty and elegance of the French language, with one describing it as “it is a beautiful language” (P11) and another highlighting their personal growth, remarking, “To have a deeper knowledge of the whole world and French-speaking realities” (P31). Cultural engagement emerged as another key intrinsic motive, reflecting a desire to connect with the language on a deeper cultural level: “I like to get to know the [French] culture” (P61). Additionally, mastering a new language was also frequently mentioned, highlighting the following: “I like learning new languages” (P87).

With respect to the (integrative motivations) interactions with French speakers, participants identified several motivational factors evolving around three main themes: ease of interaction, challenges in interaction, and lack of proficiency and confidence. Many participants reported an increasing ease of interaction over time and often linked to practice and immersion. For example, one participant stated, “I can quite easily interact with the francophones even though I’m in the process of learning French. I can understand French speaking people and I test myself when replying to them” (P17). However, challenges in interaction were also frequently mentioned, with participants pointing to difficulties in maintaining conversations or initiating them due to a lack of fluency; “I feel nervous when I speak with francophones because it takes a lot for me to make a sentence” (P99). A lack of proficiency and confidence emerged as a significant barrier to interaction. Many participants described their hesitance to engage with French speakers due to fear of making mistakes or being judged: “I can’t interact directly and easily with francophones because I hesitate to be judged because of my accent” (P81).

Regarding interest in learning other languages and cultures, many participants expressed a deep enthusiasm for learning other languages and often linked this interest to personal fulfillment: “I always want to discover other languages. I love the idea that you can talk to people from other cultures and discover them” (P55). Practical use in everyday life also emerged as a significant motivation, with participants frequently citing work, travel, and integration into communities as key reasons for learning additional languages, noting. Additionally, cultural curiosity further underpinned participants’ interest in learning languages. Many responses reflected a desire to understand and engage with diverse cultures, with one participant stating, “because speaking several languages opens up the possibility of getting to know more cultural people” (P103). This theme underscores the role of language as a bridge to cultural exploration and global awareness.

When it comes to the competition and cooperation in learning French, the majority of participants expressed a strong preference for cooperation by emphasizing its effectiveness in enhancing learning, particularly in oral practices and group activities. Learners frequently cited cooperative activities, such as dialogues, group discussions, and presentations, as instrumental in improving speaking skills and building confidence. A participant noted, “Cooperation is important because the study of a foreign language requires communication. For example, you can improve your level of oral production by doing dialogues with other students” (P59). In contrast, competition was less favored, with many participants associating it with stress and discomfort. One participant stated, “I think a competitive environment generates more stress and learning” (P109), while another remarked, “I don’t like [competition]. Learning French is not a competition. All learners have to support each other.” (P39). However, a smaller group of participants highlighted the motivational benefits of friendly competition, particularly in structured activities like vocabulary quizzes or speaking challenges: “I think friendly competition is important for better learning” (P97).

Course materials, emerging as another important theme, were seen as crucial not only for understanding grammar and vocabulary but also for improving pronunciation and cultural awareness. Among the various types of materials, audiovisual resources were the most frequently mentioned. Participants emphasized the effectiveness of videos, films, and audio materials in improving listening skills, pronunciation, and overall comprehension: “Yes, because [course materials] give us a record of what we’ve already seen and what we’ll see next. It’s a way of consulting during questions, and ultimately it’s useful because people have different ways of learning (visual, auditory, etc.)” (P45). Another participant highlighted, “Audio material is particularly important throughout the learning process. It’s the only way to improve pronunciation” (P88). Songs and music were also highlighted as engaging and enjoyable tools for language learning.

With regard to the attitudes of participants toward learning French, the majority expressed positive feelings about learning French, describing the experience as exciting, cool, and motivating. Many participants highlighted the enjoyment of discovering new expressions and cultural nuances, which made learning engaging and rewarding: “I like learning new things so it’s exciting and fun. Plus, it’s a beautiful language and since I speak Spanish, I can combine or better understand my mother tongue” (P110). However, some participants reported negative or challenging feelings related to the difficulties of learning French. These challenges were often linked to stress, frustration, and fear of making mistakes, particularly in speaking and pronunciation. For instance, one participant stated, “I feel terrible because it is difficult for me, it doesn’t resemble to my native language nor English. Grammar, pronunciation, everything is difficult for me” (P78).

For expectancy, the primary motivations for learning French were driven by academic and career-oriented goals, as well as social integration. Many participants highlighted the importance of French for academic success and career advancement and viewed it as a critical skill for securing job opportunities and excelling in professional settings. For example, one participant stated, “I’m learning French because I want to have enough knowledge to find an internship, first, and a job, second…” (P35), while another remarked, “I’m learning French because it’s one of the most widely used official languages in international organizations. I want to work for international organizations in the future” (P97). Social integration also emerged as a prominent theme, with many participants motivated by the desire to connect with local communities and navigate daily life in French-speaking environments: “I’m also learning [French] because I want to integrate fully into the city and feel comfortable living there” (P102).

Regarding language aptitude, many participants felt competent in grammar, reading comprehension, and listening skills when learning French. These areas were frequently cited as strengths and considered easier to develop compared to speaking or writing. For example, one participant noted, “Grammar and reading comprehension are the easiest for me, [whereas] speaking is the most difficult” (P41). However, about half of the participants identified pronunciation and speaking as their primary challenges. Issues with articulation, minimal pairs, and accents were commonly mentioned as barriers to fluency: “…pronunciation is still a problem because there are minimal pairs that aren’t in Spanish, so my ear hasn’t completely got used to it yet and it’s mainly my oral production that poses problems with articulation, these minimal pairs and my accent” (P64).

For the question which asked whether the participants are anxious or relaxed in regard to learning French, many participants experienced significant anxiety while learning French, particularly during specific activities, such as speaking, exams, and real-life interactions. Speaking emerged as the most prominent source of anxiety, often attributed to a lack of confidence, fear of making mistakes, and concerns about being misunderstood. One participant stated, “I am anxious when I have to speak because I lack confidence when I express myself in French” (P07), while another participant commented, “I am relaxing, but during exam I am anxious I am not confident with my oral speaking” (P26). In contrast, few participants reported feeling relaxed during structured and predictable learning environments: “Now I’m more relaxed because I’m better able to understand it [in French] both orally and in written expression” (P77).

With respect to the intention to put his/her best effort into learning French, most participants expressed a strong intention to continue learning French, driven by professional and career goals, social integration, and personal enrichment. Career advancement was a prominent theme, emphasizing the importance of French for job opportunities and professional fluency “… because knowing French has important consequences in my personal and professional life” (P111), while another remarked, “I will do my master’s in France or work there in the future” (P47). Social integration also emerged as a significant motive, highlighting the need to communicate effectively and build relationships in French-speaking communities. One participant shared, “because I want to integrate well into the French-speaking environment” (P99). Additionally, many participants expressed a deep passion for the French language for their personal enrichment, emphasizing; “I want to be fluent and speak like a native” (P85).

## 6. Discussion

This study investigated the motivations of multilingual university students in learning French as an additional language and LOTE within the Belgian context. Regarding the differences in various motivational constructs related to learning French (RQ1), the results indicated that multilingual university students are influenced by a diverse set of motivational factors, each contributing meaningfully to their overall motivation. Notably, the particularly high ratings for interest in foreign languages and cultures, alongside cooperativeness, underscore the central role of intrinsic curiosity and socially driven dispositions in sustaining multilingual learners’ engagement with additional language learning. These findings align with both the socio-educational model and self-determination theory in fostering L2 learners’ engagement (Ryan & Deci, 2002; Gardner, 2010). In multilingual contexts, these same motivational components remain valid, playing a crucial role in supporting multilingual learners’ acquisition of additional languages and LOTE. Although competitiveness and anxiety yielded lower mean scores, their statistical significance indicates that they remain relevant factors within the broader motivational landscape. These findings underscore that multilingual learners’ motivation for additional language learning and LOTE is shaped by a dynamic interplay of personal dispositions and contextual influences, reflecting the complex and evolving nature of multilingual motivation. Additionally, the variation in motivational constructs among multilingual learners aligns with previous research suggesting that language-learning motivation is dynamic and influenced by learners’ linguistic backgrounds, personal goals, and sociocultural contexts (Bui et al., 2018; Lai, 2023; Siridetkoon & Dewaele, 2018).

In relation to gender differences in motivational constructs (RQ2), we found no statistically significant variations between male and female learners in their motivation to learn French. This result suggests that multilingual male and female French learners exhibit comparable motivational profiles in the context of learning French as LOTE. This finding also aligns with previous research, which has similarly reported no gender-based difference in language-learning motivation (Henry & Cliffordson, 2013; Sylvén & Thompson, 2015; Thompson & Erdil-Moody, 2016).

With regard to the relationship between motivation and language proficiency (RQ2), the present study found that multilingual learners of French shared consistent motivational patterns across a broad range of constructs, including intrinsic and extrinsic motivation, interest in languages and culture, integrative motivation, competitiveness, cooperativeness, the value of the language course materials, the belief in doing well or getting high grades, attitudes toward the target language, and overall motivational strengths. These results suggest that motivation to learn French remains relatively stable across different levels of language proficiency. However, significant differences were observed in language aptitude and language-learning anxiety, with advanced learners (B2–C1) reporting higher aptitude and lower anxiety than learners at lower proficiency levels (A2–B1) (Table 3). Accordingly, multilingual advanced French learners demonstrated stronger language aptitude and reported significantly lower anxiety levels, suggesting that as learners become more proficient, they develop greater confidence and reduced affective barriers. Previous research also supports these findings, indicating that lower levels of anxiety are often associated with higher language proficiency and more clearly defined motivational trajectories in multilingual learning contexts, with similar patterns also observed in L2 learning (Csizér & Kormos, 2009; Teimouri et al., 2019; Thompson & Erdil-Moody, 2016).

The findings related to participants’ specific motivational factors for learning French (RQ3) provide valuable insights into the multifaceted motivations and contextual influences that shape multilingual university students’ experiences. Career advancement, academic success, and social integration emerged as dominant extrinsic drivers of multilingual university students in learning French. Given that French was the medium of instruction for the participants in their university study programs, these motives might be particularly salient extrinsic motivators, shaping multilingual students’ positive attitudes toward learning French as a means to succeed in their academic programs. Additionally, extrinsic motivation related to academic and professional advancement is widely supported in recent multilingualism research (Lamb et al., 2019; Costache et al., 2022). These motivations are closely linked to the ought-to L2 self in L2MSS, which represents external pressures and expectations imposed by the learner’s environment (Dörnyei & Al-Hoorie, 2017). Additionally, the finding related to social integration aligns with Gardner’s (1985) concept of integrative motivation, which emphasizes learners’ desire to connect with and become part of the target language community. In addition to extrinsic motivational drives, the results also indicated that participants were intrinsically motivated by their enjoyment of the aesthetics of the French language. They described a personal interest in French culture, an appreciation for the language’s beauty, and a desire for self-development. The results align with SDT, which emphasizes the role of autonomous motivation, where learners engage in language study for reasons of personal interest or inherent satisfaction (Ryan & Deci, 2017; Noels et al., 2000). Moreover, the participants’ aspirations resonate with Dörnyei’s (2009) concept of the Ideal L2 Self, reflecting a vision of themselves as proficient French speakers and culturally engaged individuals in multilingual context, which serves as a powerful internal motivator in sustaining language-learning effort. Furthermore, participants’ desire to explore Francophone culture aligns with Ushioda’s (2011) view of motivation as relational and identity-driven, grounded in learners’ cultural and personal values.

At the same time, learners’ experiences with learning French varied widely. While some multilingual students reported increased ease and confidence through practice and immersion, others highlighted the challenges of oral communication, particularly due to fear of making mistakes, language accent, etc. These support previous findings that multilingual learners, while often more linguistically flexible, may still face emotional barriers when acquiring an additional language (Dewaele et al., 2008; Thompson & Erdil-Moody, 2016). In addition, anxiety emerged prominently among participants, particularly concerning oral skills, accent, pronunciation, and fear of negative evaluation in learning French. Participants at higher proficiency levels reported increased confidence and reduced anxiety, aligning with previous findings that highlight significant variations in anxiety across different proficiency levels (Teimouri et al., 2019; Thompson & Erdil-Moody, 2016). However, multilingual learners expressed positive attitudes toward learning French, describing the experience as enjoyable and enriching. Less proficient learners often reported feelings of frustration and anxiety, particularly related to challenges with grammar, pronunciation, and fluency. These findings are consistent with Dewaele and MacIntyre’s (2016) findings, which emphasize the coexistence of enjoyment and anxiety in language learning across different proficiency levels.

When it comes to learning environments, the findings indicate that almost all participants favored cooperative learning strategies over competition. Cooperative learning activities, including group discussions and pair work, in multilingual learning settings were perceived to enhance speaking ability and reduce anxiety. This finding is consistent with previous research (Bećirović, 2023) that reflects the “learning experience” component of Dörnyei’s (2009) L2MSS, highlighting how cooperative classroom dynamics positively influence motivation by fostering engagement, enhancing speaking confidence, and reducing anxiety. In terms of expectancy, multilingual university students were primarily motivated by the perceived benefits of learning French for future career opportunities, academic advancement, and social integration. In a context where French served as the medium of instruction for the participants’ study programs, these motives might serve as key factors motivating multilingual learners to acquire French. Such extrinsically driven motivations align with the ought-to L2 self in L2MSS, which reflects the external expectations, obligations and social pressures perceived by learners in their academic and professional environments (Dörnyei & Al-Hoorie, 2017).

The results also highlighted participants’ emphasis on the importance of instructional materials, particularly audiovisual content, for enhancing comprehension, pronunciation, and cultural understanding. In terms of goal orientation and effort, most learners reported strong intentions to continue learning French, driven by professional, academic, and social-integration goals. This aligns with the Ideal L2 Self, where learners envision themselves as successful language users, and is especially prominent in multilingual individuals (Dörnyei & Al-Hoorie, 2017; Thompson & Erdil-Moody, 2016; Henry, 2017). Learners’ strong ambitions to use French in their future careers or lives abroad also highlight this internally driven motivational path (Dörnyei, 2009; Ryan & Deci, 2017).

Finally, learners’ perceptions of language aptitude varied. Many felt more competent in receptive skills like reading and listening, while speaking and pronunciation were often cited as the most difficult. Notably, multilingual learners frequently reported drawing on their prior language-learning experiences which is closely consistent with the view of the “learning experience” in L2MSS (Dörnyei, 2009). This also aligns with the perspectives of Cenoz (2013) and Cummins (2007), who emphasize that multilinguals transfer metalinguistic awareness and strategic learning resources across languages.

## 7. Conclusions

This study explored the complex and dynamic motivational landscape of multilingual university students learning French as an additional language and LOTE. The findings revealed that motivation for additional language and LOTE learning among multilinguals is multifaceted, shaped primarily by a dynamic interplay of intrinsic and extrinsic factors, integrative motives, and prior learning experiences. Multilingual university students were found to be motivated not only by extrinsic goals, such as academic success and career advancement, but also by intrinsic interests, including personal growth, cultural appreciation, and aesthetic enjoyment of the French language. Additionally, social integration and prior language-learning experiences emerged as key motivational factors, highlighting the importance of both relational and experiential influences in shaping multilingual learners’ engagement with French. These motivational drivers among the multilingual learners also align with major theoretical frameworks in L2 motivation, including Gardner’s Socio-Educational model (Gardner, 1985, 2010), SDT (Deci & Ryan, 1985; Ryan & Deci, 2017), and Dörnyei’s L2MSS (Dörnyei, 2005, 2009). In addition, this study also revealed that multilingual learners demonstrated distinct and multifaceted motivational patterns when engaging with additional languages and LOTE. These patterns reflect the complexity of their multilingual identities and are influenced by a blend of personal, emotional, and contextual factors (Cenoz, 2003; Costache et al., 2022; Dörnyei & Ushioda, 2009). While some motivations are specific to the target language—such as career development, cultural affinity, or personal interest in French—others are shaped by broader multilingual experiences of prior language learning (Dörnyei & Al-Hoorie, 2017).

## 8. Practical Implications, Limitations, and Future Directions

This study practically contributes to a broader understanding of the complex relationships among additional language-learning, motivation, and multilingual learners, offering pedagogical implications for more inclusive and effective language education in multilingual settings. Given the diverse motivational patterns identified among multilingual learners, educators might offer flexible learning pathways that allow students to pursue their individual interests, such as content-based instruction linking target language learning with relevant themes like art, history, or global issues. Additionally, to foster integrative motivation, educators could create meaningful opportunities for interaction both within and beyond the classroom, thereby enhancing learners’ social integration and promoting sustained engagement in additional language and LOTE learning. Moreover, language policies at the university could move beyond monolingual paradigms and embrace the dynamic multilingual identities of students. Policymakers, in collaboration with educational institutions, could develop and implement policies that support linguistic diversity, value proficiency in multiple languages, and recognize multilingualism as an asset in both academic and professional contexts.

While this study provides significant insights, it also presents certain limitations. It primarily relied on self-reported data, which may have been influenced by subjective perceptions or social desirability effects. Future research could benefit from longitudinal designs to explore how multilingual learners’ motivations develop and shift over time, offering a more nuanced understanding of motivational trajectories. Additionally, experimental studies could be conducted to evaluate the effectiveness of targeted interventions on additional language-learning and LOTE motivation within multilingual contexts. Moreover, further studies among multilingual learners in various linguistic and educational contexts would enrich our understanding of motivational interrelationships and their implications across diverse learner populations. It is also important to note that this research included multilingual learners across all proficiency levels, without a specific focus on one. Future research could explore and compare various experiences and motivational profiles of learners at specific proficiency levels within multilingual contexts.

## Figures and Tables

**Table 1:** Distribution of participants’ mother tongues (L1).

Mother Tongues (L1)	N	%
Albanian	2	1.7
Arabic	6	5.0
Bulgarian	1	0.8
Chinese	6	5.0
Czech	3	2.5
English	4	3.3
Estonian	1	0.8
Finnish	1	0.8
German	13	10.7
Greek	1	0.8
Indian	1	0.8
Italian	27	22.3
Japanese	1	0.8
Korean	4	3.3
Persian	1	0.8
Polish	3	2.5
Portuguese	5	4.1
Romanian	4	3.3
Slovak	1	0.8
Spanish	15	12.4
Turkish	16	13.2
Ukrainian	4	3.3
Vietnamese	1	0.8
Total	121	100.0

**Table 2:** Means of various motivational constructs in learning French.

Motivational Constructs	(n = 121) Mean (SD)
Intrinsic motivation	3.66 (0.54)
Extrinsic motivation	3.69 (0.53)
Integrative motivation	3.81 (0.70)
Interest in foreign language and culture	4.18 (0.52)
Competitiveness	2.76 (1.12)
Cooperativeness	3.98 (0.59)
Task-value	3.82 (0.70)
Expectancy	3.61 (0.50)
Language aptitude	3.26 (0.69)
Attitude	3.00 (0.63)
Anxiety	2.71 (0.80)
Motivational strengths	3.71 (0.47)

**Table 3:** One-Way ANOVA results on motivational constructs in learning French based on students’ proficiency levels.

Motivational Constructs	Proficiency Levels	N	X	SD	df	F	*p*
Intrinsic motivation	A2-Elementary	17	3.67	0.491			
	B1-Intermediate	46	3.61	0.530	3	1.243	0.297
	B2-Upper-Intermediate	45	3.77	0.502			
	C1-Advanced	13	3.47	0.790			
Extrinsic motivation	A2-Elementary	17	3.59	0.506			
	B1-Intermediate	46	3.62	0.495	3	1.257	0.292
	B2-Upper-Intermediate	45	3.81	0.549			
	C1-Advanced	13	3.65	0.612			
Integrative motivation	A2-Elementary	17	3.79	0.601			
	B1-Intermediate	46	3.80	0.699	3	0.164	0.920
	B2-Upper-Intermediate	45	3.86	0.696			
	C1-Advanced	13	3.71	0.894			
Interest in foreign language and culture	A2-Elementary	17	3.92	0.628			
	B1-Intermediate	46	4.21	0.489	3	1.824	0.147
	B2-Upper-Intermediate	45	4.26	0.451			
	C1-Advanced	13	4.15	0.669			
Competitiveness	A2-Elementary	17	2.84	0.965			
	B1-Intermediate	46	2.65	1.073	3	0.762	0.518
	B2-Upper-Intermediate	45	2.93	1.236			
	C1-Advanced	13	2.48	1.085			
Cooperativeness	A2-Elementary	17	3.80	0.681	3	1.311	0.274
	B1-Intermediate	46	4.00	0.574			
	B2-Upper-Intermediate	45	4.08	0.598			
	C1-Advanced	13	3.80	0.501			
Task-value	A2-Elementary	17	3.64	0.594			
	B1-Intermediate	46	3.76	0.719	3	1.040	0.378
	B2-Upper-Intermediate	45	3.96	0.689			
	C1-Advanced	13	3.82	0.812			
Expectancy	A2-Elementary	17	3.45	0.516			
	B1-Intermediate	46	3.65	0.419	3	1.495	0.219
	B2-Upper-Intermediate	45	3.68	0.586			
	C1-Advanced	13	3.44	0.410			
Language aptitude	A2-Elementary	17	2.82	0.513			
	B1-Intermediate	46	3.21	0.716	3	3.716	0.013
	B2-Upper-Intermediate	45	3.40	0.681			
	C1-Advanced	13	3.51	0.680			
Attitude	A2-Elementary	17	3.01	0.676			
	B1-Intermediate	46	2.96	0.712	3	0.833	0.478
	B2-Upper-Intermediate	45	3.10	0.547			
	C1-Advanced	13	2.81	0.556			
Anxiety	A2-Elementary	17	3.00	0.793			
	B1-Intermediate	46	2.93	0.848	3	4.453	0.005
	B2-Upper-Intermediate	45	2.50	0.730			
	C1-Advanced	13	2.30	0.588			
Motivational strengths	A2-Elementary	17	3.89	0.372			
	B1-Intermediate	46	3.69	0.394	3	1.918	0.131
	B2-Upper-Intermediate	45	3.73	0.534			
	C1-Advanced	13	3.48	0.564			

## Data Availability

The data that support the findings of this study are available upon request from the corresponding author. The data are not publicly available due to privacy or ethical restrictions.
